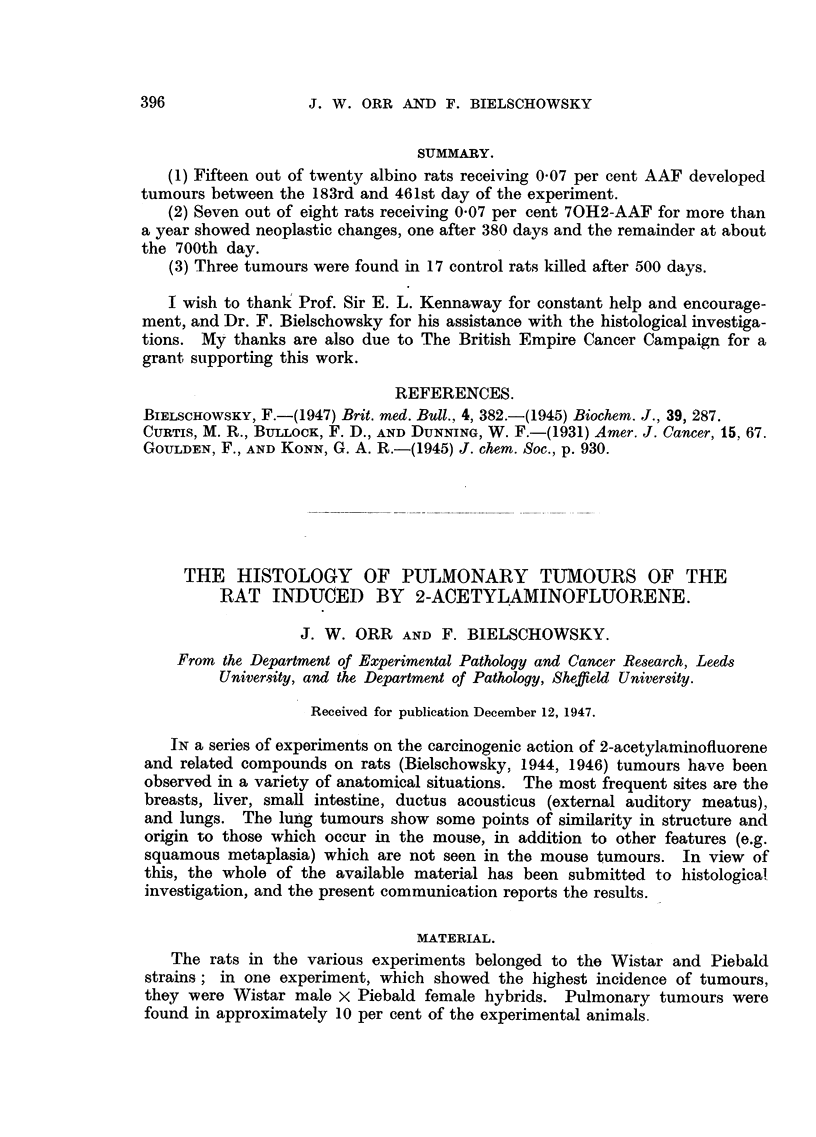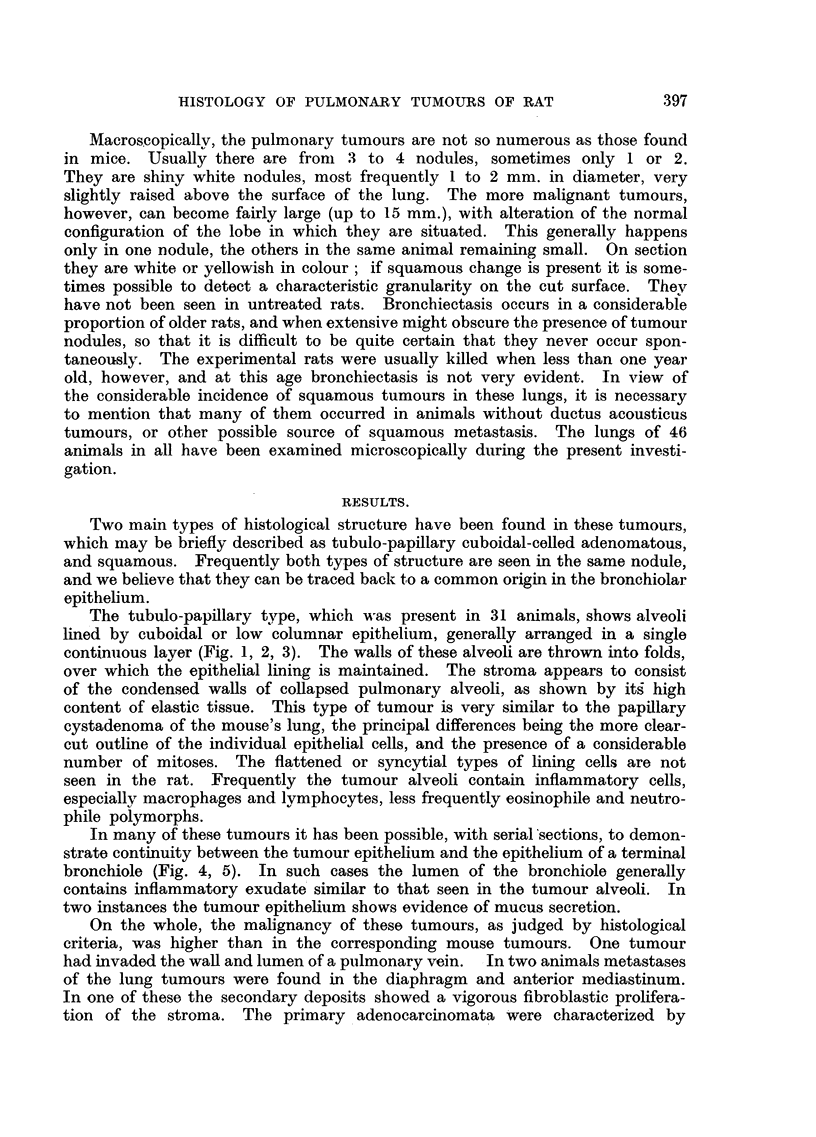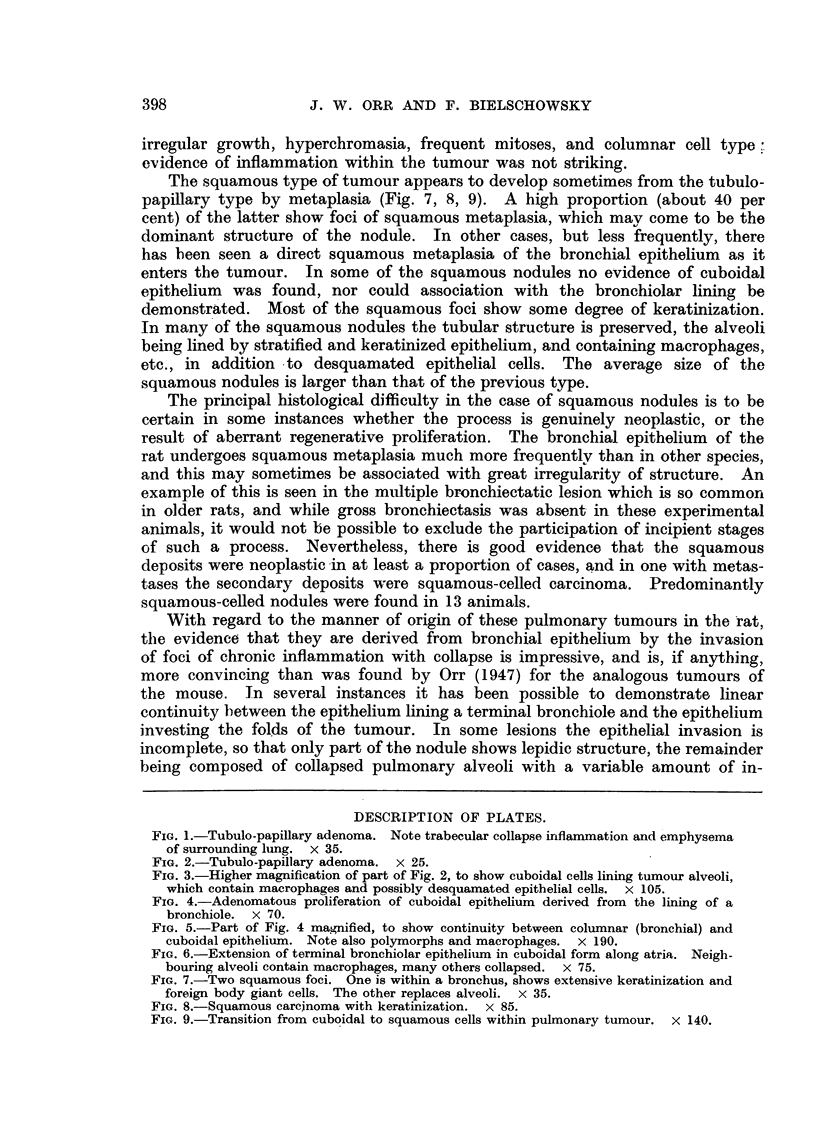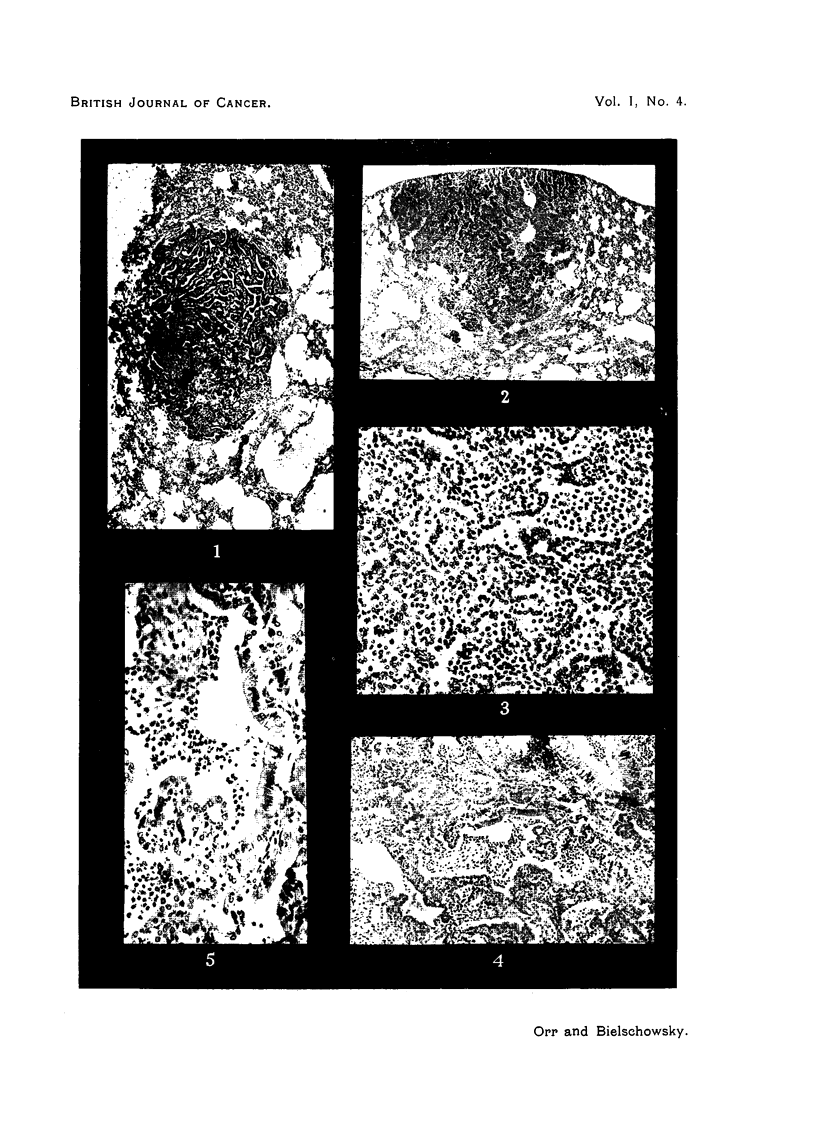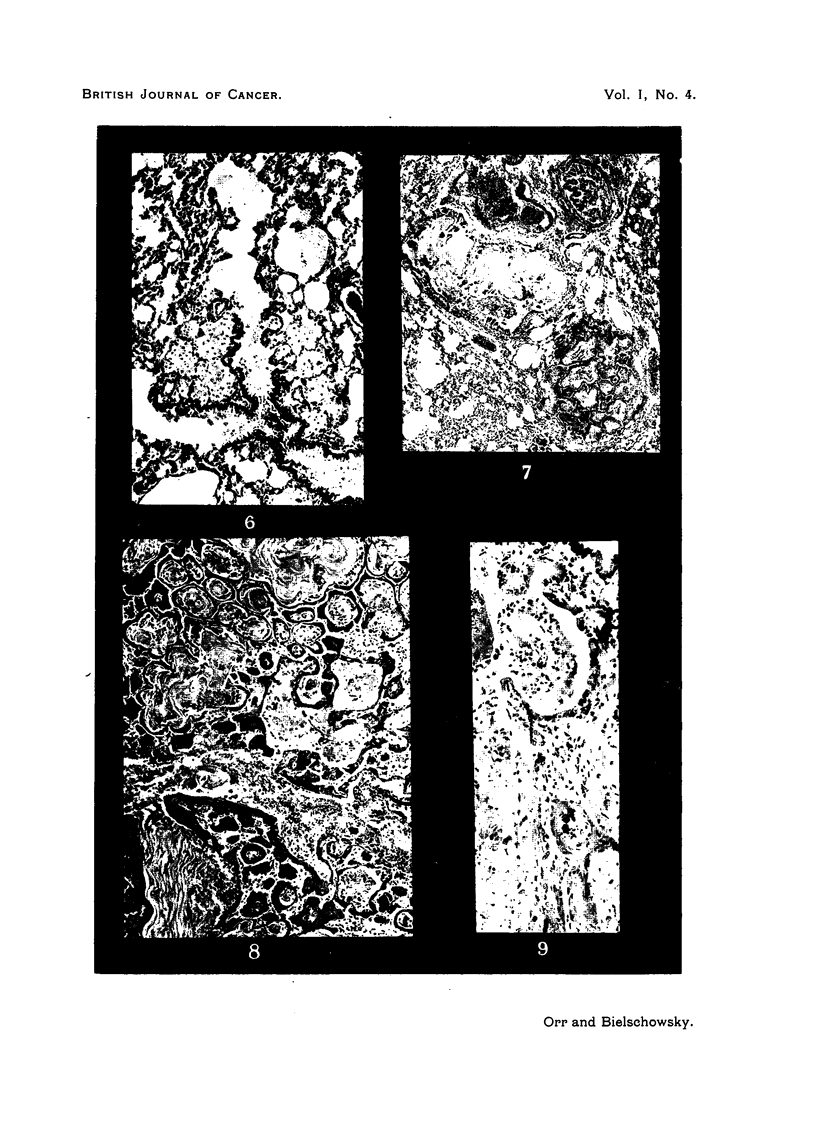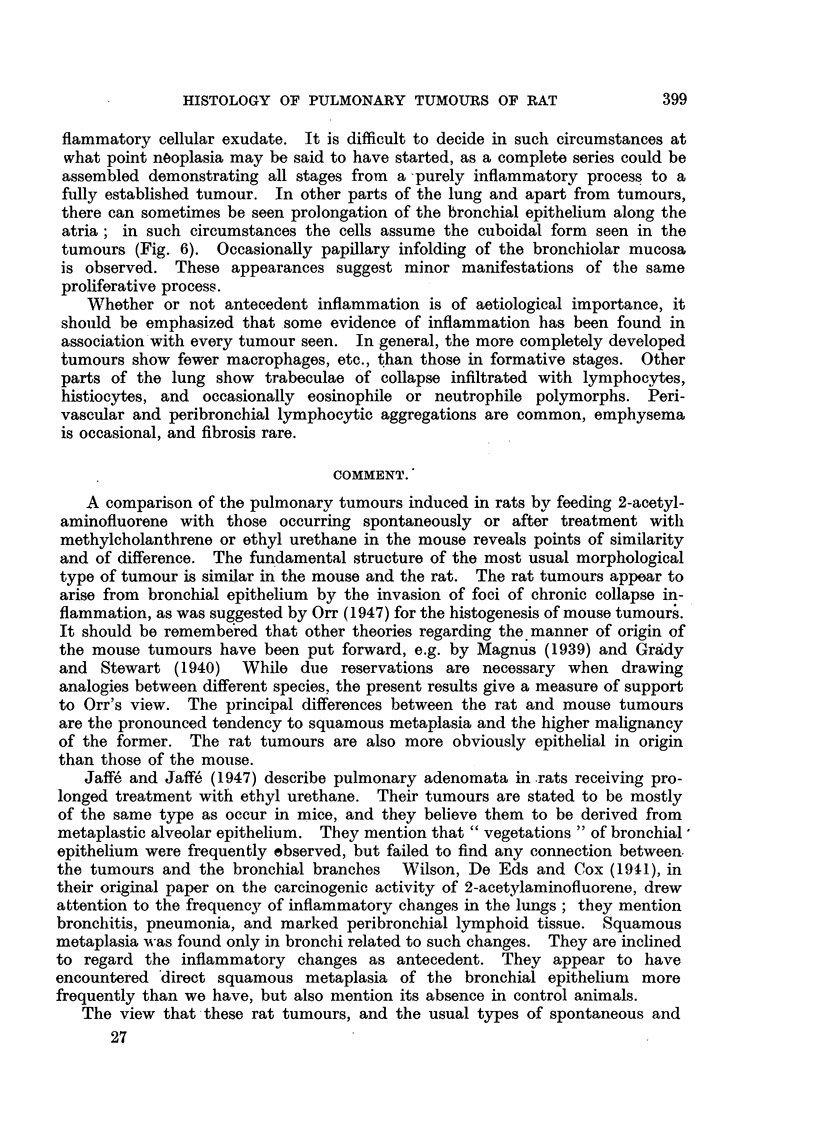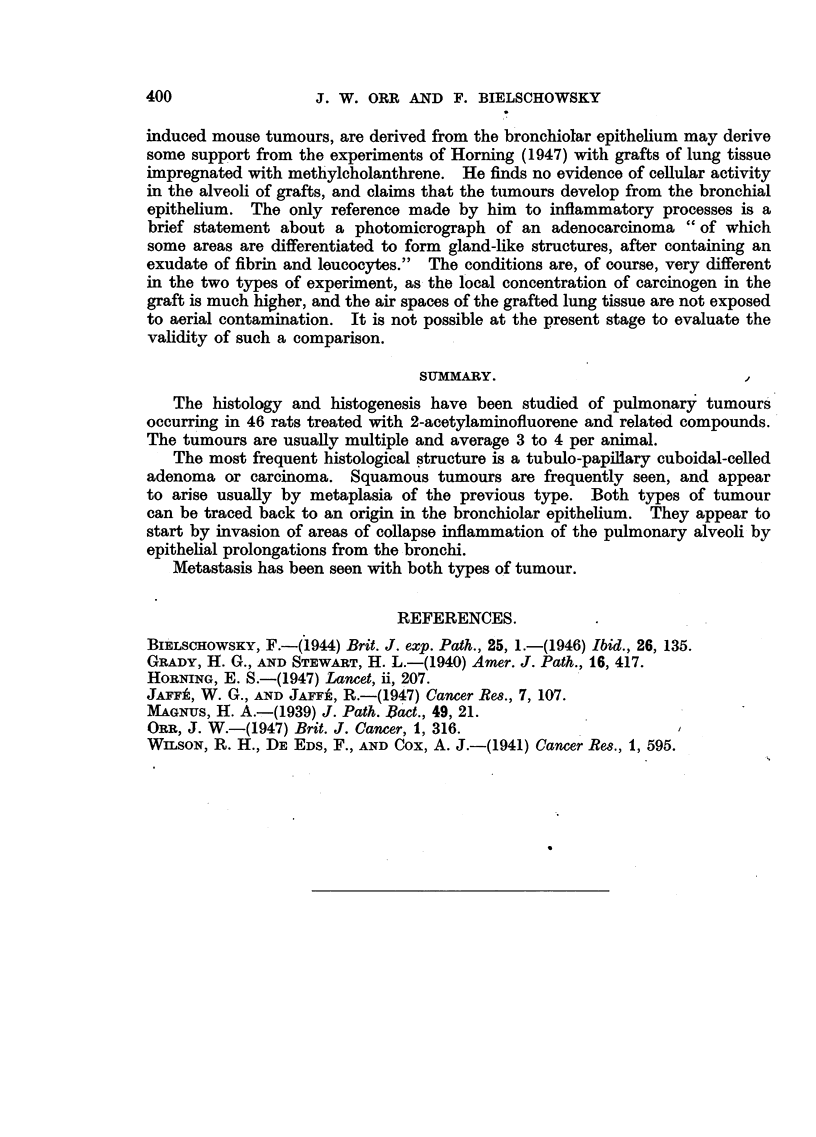# The Histology of Pulmonary Tumours of the Rat Induced by 2-Acetylaminofluorene

**DOI:** 10.1038/bjc.1947.38

**Published:** 1947-12

**Authors:** J. W. Orr, F. Bielschowsky

## Abstract

**Images:**


### THE HISTOLOGY OF PULMONARY TUMOURS OF THE RAT INDUCED BY 2-ACETYLAMINOFLUORENE.

J. W. ORR and F. BIELSCHOWSKY.

*From the Department of Experimental Pathology and Cancer Research, Leeds University, and the Department of Pathology, Sheffield University.*

Received for publication December 12, 1947.

In a series of experiments on the carcinogenic action of 2-acetylaminofluorene and related compounds on rats (Bielschowsky, 1944, 1946) tumours have been observed in a variety of anatomical situations. The most frequent sites are the breasts, liver, small intestine, ductus acousticus (external auditory meatus), and lungs. The lung tumours show some points of similarity in structure and origin to those which occur in the mouse, in addition to other features (e.g. squamous metaplasia) which are not seen in the mouse tumours. In view of this, the whole of the available material has been submitted to histological investigation, and the present communication reports the results.

### MATERIAL.

The rats in the various experiments belonged to the Wistar and Piebald strains; in one experiment, which showed the highest incidence of tumours, they were Wistar male × Piebald female hybrids. Pulmonary tumours were found in approximately 10 per cent of the experimental animals.

Macroscopically, the pulmonary tumours are not so numerous as those found in mice. Usually there are from 3 to 4 nodules, sometimes only 1 or 2. They are shiny white nodules, most frequently 1 to 2 mm. in diameter, very slightly raised above the surface of the lung. The more malignant tumours, however, can become fairly large (up to 15 mm.), with alteration of the normal configuration of the lobe in which they are situated. This generally happens only in one nodule, the others in the same animal remaining small. On section they are white or yellowish in colour ; if squamous change is present it is sometimes possible to detect a characteristic granularity on the cut surface. They have not been seen in untreated rats. Bronchiectasis occurs in a considerable proportion of older rats, and when extensive might obscure the presence of tumour nodules, so that it is difficult to be quite certain that they never occur spontaneously. The experimental rats were usually killed when less than one year old, however, and at this age bronchiectasis is not very evident. In view of the considerable incidence of squamous tumours in these lungs, it is necessary to mention that many of them occurred in animals without ductus acousticus tumours, or other possible source of squamous metastasis. The lungs of 46 animals in all have been examined microscopically during the present investigation.

### RESULTS.

Two main types of histological structure have been found in these tumours, which may be briefly described as tubulo-papillary cuboidal-celled adenomatous, and squamous. Frequently both types of structure are seen in the same nodule, and we believe that they can be traced back to a common origin in the bronchiolar epithelium.

The tubulo-papillary type, which was present in 31 animals, shows alveoli lined by cuboidal or low columnar epithelium, generally arranged in a single continuous layer (Fig. 1, 2, 3). The walls of these alveoli are thrown into folds, over which the epithelial lining is maintained. The stroma appears to consist of the condensed walls of collapsed pulmonary alveoli, as shown by its high content of elastic tissue. This type of tumour is very similar to the papillary cystadenoma of the mouse's lung, the principal differences being the more clear-cut outline of the individual epithelial cells, and the presence of a considerable number of mitoses. The flattened or syncytial types of lining cells are not seen in the rat. Frequently the tumour alveoli contain inflammatory cells, especially macrophages and lymphocytes, less frequently eosinophile and neutrophile polymorphs.

In many of these tumours it has been possible, with serial sections, to demonstrate continuity between the tumour epithelium and the epithelium of a terminal bronchiole (Fig. 4, 5). In such cases the lumen of the bronchiole generally contains inflammatory exudate similar to that seen in the tumour alveoli. In two instances the tumour epithelium shows evidence of mucus secretion.

On the whole, the malignancy of these tumours, as judged by histological criteria, was higher than in the corresponding mouse tumours. One tumour had invaded the wall and lumen of a pulmonary vein. In two animals metastases of the lung tumours were found in the diaphragm and anterior mediastinum. In one of these the secondary deposits showed a vigorous fibroblastic proliferation of the stroma. The primary adenocarcinomata were characterized by irregular growth, hyperchromasia, frequent mitoses, and columnar cell type: evidence of inflammation within the tumour was not striking.

The squamous type of tumour appears to develop sometimes from the tubulo-papillary type by metaplasia (Fig. 7, 8, 9). A high proportion (about 40 per cent) of the latter show foci of squamous metaplasia, which may come to be the dominant structure of the nodule. In other cases, but less frequently, there has been seen a direct squamous metaplasia of the bronchial epithelium as it enters the tumour. In some of the squamous nodules no evidence of cuboidal epithelium was found, nor could association with the bronchiolar lining be demonstrated. Most of the squamous foci show some degree of keratinization. In many of the squamous nodules the tubular structure is preserved, the alveoli being lined by stratified and keratinized epithelium, and containing macrophages, etc., in addition to desquamated epithelial cells. The average size of the squamous nodules is larger than that of the previous type.

The principal histological difficulty in the case of squamous nodules is to be certain in some instances whether the process is genuinely neoplastic, or the result of aberrant regenerative proliferation. The bronchial epithelium of the rat undergoes squamous metaplasia much more frequently than in other species, and this may sometimes be associated with great irregularity of structure. An example of this is seen in the multiple bronchiectatic lesion which is so common in older rats, and while gross bronchiectasis was absent in these experimental animals, it would not be possible to exclude the participation of incipient stages of such a process. Nevertheless, there is good evidence that the squamous deposits were neoplastic in at least a proportion of cases, and in one with metastases the secondary deposits were squamous-celled carcinoma. Predominantly squamous-celled nodules were found in 13 animals.

With regard to the manner of origin of these pulmonary tumours in the rat, the evidence that they are derived from bronchial epithelium by the invasion of foci of chronic inflammation with collapse is impressive, and is, if anything, more convincing than was found by Orr (1947) for the analogous tumours of the mouse. In several instances it has been possible to demonstrate linear continuity between the epithelium lining a terminal bronchiole and the epithelium investing the folds of the tumour. In some lesions the epithelial invasion is incomplete, so that only part of the nodule shows lepidic structure, the remainder being composed of collapsed pulmonary alveoli with a variable amount of in-

---

DESCRIPTION OF PLATES.

FIG. 1.—Tubulo-papillary adenoma. Note trabecular collapse inflammation and emphysema of surrounding lung. × 35.

FIG. 2.—Tubulo-papillary adenoma. × 25.

FIG. 3.—Higher magnification of part of Fig. 2, to show cuboidal cells lining tumour alveoli, which contain macrophages and possibly desquamated epithelial cells. × 105.

FIG. 4.—Adenomatous proliferation of cuboidal epithelium derived from the lining of a bronchiole. × 70.

FIG. 5.—Part of Fig. 4 magnified, to show continuity between columnar (bronchial) and cuboidal epithelium. Note also polymorphs and macrophages. × 190.

FIG. 6.—Extension of terminal bronchiolar epithelium in cuboidal form along atria. Neighbouring alveoli contain macrophages, many others collapsed. × 75.

FIG. 7.—Two squamous foci. One is within a bronchus, shows extensive keratinization and foreign body giant cells. The other replaces alveoli. × 35.

FIG. 8.—Squamous carcinoma with keratinization. × 85.

FIG. 9.—Transition from cuboidal to squamous cells within pulmonary tumour. × 140.

1

2

3

4

5

Orr and Bielschowsky.

6 7 8 9

Orr and Bielschowsky.

flammatory cellular exudate. It is difficult to decide in such circumstances at what point neoplasia may be said to have started, as a complete series could be assembled demonstrating all stages from a purely inflammatory process to a fully established tumour. In other parts of the lung and apart from tumours, there can sometimes be seen prolongation of the bronchial epithelium along the atria ; in such circumstances the cells assume the cuboidal form seen in the tumours (Fig. 6). Occasionally papillary infolding of the bronchiolar mucosa is observed. These appearances suggest minor manifestations of the same proliferative process.

Whether or not antecedent inflammation is of aetiological importance, it should be emphasized that some evidence of inflammation has been found in association with every tumour seen. In general, the more completely developed tumours show fewer macrophages, etc., than those in formative stages. Other parts of the lung show trabeculae of collapse infiltrated with lymphocytes, histiocytes, and occasionally eosinophile or neutrophile polymorphs. Perivascular and peribronchial lymphocytic aggregations are common, emphysema is occasional, and fibrosis rare.

### COMMENT.

A comparison of the pulmonary tumours induced in rats by feeding 2-acetylaminofluorene with those occurring spontaneously or after treatment with methylcholanthrene or ethyl urethane in the mouse reveals points of similarity and of difference. The fundamental structure of the most usual morphological type of tumour is similar in the mouse and the rat. The rat tumours appear to arise from bronchial epithelium by the invasion of foci of chronic collapse inflammation, as was suggested by Orr (1947) for the histogenesis of mouse tumours. It should be remembered that other theories regarding the manner of origin of the mouse tumours have been put forward, e.g. by Magnus (1939) and Grady and Stewart (1940) While due reservations are necessary when drawing analogies between different species, the present results give a measure of support to Orr's view. The principal differences between the rat and mouse tumours are the pronounced tendency to squamous metaplasia and the higher malignancy of the former. The rat tumours are also more obviously epithelial in origin than those of the mouse.

Jaffé and Jaffé (1947) describe pulmonary adenomata in rats receiving prolonged treatment with ethyl urethane. Their tumours are stated to be mostly of the same type as occur in mice, and they believe them to be derived from metaplastic alveolar epithelium. They mention that " vegetations " of bronchial epithelium were frequently observed, but failed to find any connection between the tumours and the bronchial branches Wilson, De Eds and Cox (1941), in their original paper on the carcinogenic activity of 2-acetylaminofluorene, drew attention to the frequency of inflammatory changes in the lungs ; they mention bronchitis, pneumonia, and marked peribronchial lymphoid tissue. Squamous metaplasia was found only in bronchi related to such changes. They are inclined to regard the inflammatory changes as antecedent. They appear to have encountered direct squamous metaplasia of the bronchial epithelium more frequently than we have, but also mention its absence in control animals.

The view that these rat tumours, and the usual types of spontaneous and induced mouse tumours, are derived from the bronchiolar epithelium may derive some support from the experiments of Horning (1947) with grafts of lung tissue impregnated with methylcholanthrene. He finds no evidence of cellular activity in the alveoli of grafts, and claims that the tumours develop from the bronchial epithelium. The only reference made by him to inflammatory processes is a brief statement about a photomicrograph of an adenocarcinoma "of which some areas are differentiated to form gland-like structures, after containing an exudate of fibrin and leucocytes." The conditions are, of course, very different in the two types of experiment, as the local concentration of carcinogen in the graft is much higher, and the air spaces of the grafted lung tissue are not exposed to aerial contamination. It is not possible at the present stage to evaluate the validity of such a comparison.

### SUMMARY.

The histology and histogenesis have been studied of pulmonary tumours occurring in 46 rats treated with 2-acetylaminofluorene and related compounds. The tumours are usually multiple and average 3 to 4 per animal.

The most frequent histological structure is a tubulo-papillary cuboidal-celled adenoma or carcinoma. Squamous tumours are frequently seen, and appear to arise usually by metaplasia of the previous type. Both types of tumour can be traced back to an origin in the bronchiolar epithelium. They appear to start by invasion of areas of collapse inflammation of the pulmonary alveoli by epithelial prolongations from the bronchi.

Metastasis has been seen with both types of tumour.

### REFERENCES.

BIELSCHOWSKY, F.—(1944) *Brit. J. exp. Path.*, **25**, 1.—(1946) *Ibid.*, **26**, 135.
GRADY, H. G., AND STEWART, H. L.—(1940) *Amer. J. Path.*, **16**, 417.
HORNING, E. S.—(1947) *Lancet*, ii, 207.
JAFFÉ, W. G., AND JAFFÉ, R.—(1947) *Cancer Res.*, **7**, 107.
MAGNUS, H. A.—(1939) *J. Path. Bact.*, **49**, 21.
ORR, J. W.—(1947) *Brit. J. Cancer*, **1**, 316.
WILSON, R. H., DE EDS, F., AND COX, A. J.—(1941) *Cancer Res.*, **1**, 595.